# Modulation of plant acetyl-CoA synthetase activity by post-translational lysine acetylation

**DOI:** 10.3389/fmolb.2023.1117921

**Published:** 2023-03-16

**Authors:** Naazneen Sofeo, Dirk C. Winkelman, Karina Leung, Basil J. Nikolau

**Affiliations:** ^1^ Roy J. Carver Department of Biochemistry, Biophysics and Molecular Biology, Iowa State University, Ames, IA, United States; ^2^ Engineering Research Center for Biorenewable Chemicals Iowa State University, Ames, IA, United States; ^3^ Center for Metabolic Biology, Iowa State University, Ames, IA, United States

**Keywords:** acetyl-CoA synthetase, acetylation, arabidopsis, genetic code expansion, post-translation modification, regulation

## Abstract

Acetyl-CoA synthetase (ACS) is one of several enzymes that generate the key metabolic intermediate, acetyl-CoA. In microbes and mammals ACS activity is regulated by the post-translational acetylation of a key lysine residue. ACS in plant cells is part of a two-enzyme system that maintains acetate homeostasis, but its post-translational regulation is unknown. This study demonstrates that the plant ACS activity can be regulated by the acetylation of a specific lysine residue that is positioned in a homologous position as the microbial and mammalian ACS sequences that regulates ACS activity, occurring in the middle of a conserved motif, near the carboxyl-end of the protein. The inhibitory effect of the acetylation of residue Lys-622 of the Arabidopsis ACS was demonstrated by site-directed mutagenesis of this residue, including its genetic substitution with the non-canonical N-ε-acetyl-lysine residue. This latter modification lowered the catalytic efficiency of the enzyme by a factor of more than 500-fold. Michaelis-Menten kinetic analysis of the mutant enzyme indicates that this acetylation affects the first half-reaction of the ACS catalyzed reaction, namely, the formation of the acetyl adenylate enzyme intermediate. The post-translational acetylation of the plant ACS could affect acetate flux in the plastids and overall acetate homeostasis.

## 1 Introduction

The interplay between acetylation and deacetylation of protein lysine residues is a crucial process in regulating many biological processes including chromatin structure, transcriptional regulation and the regulation of cellular metabolism (Bernal et al., 2014; Verdin and Ott, 2015; Drazic et al., 2016). This post-translational modification mechanism is evolutionarily conserved over a wide range of phyla, and the two enzymes that play a role in this process are: a) lysine acetyl transferases (KATs) that acetylate lysine residues; and b) lysine deacetylases (KDACs) that hydrolyze and remove the acetyl group from the side chain (Drazic et al., 2016). In addition, auto-acetylation of lysine residues occurs at alkaline pH, particularly of lysine residues that are flanked by positively charged amino acids (Narita et al., 2019).

Acetyl-CoenzymeA (CoA) synthetase (ACS) is a ∼72 kDa protein that catalyzes the synthesis of acetyl-CoA from acetate and in microbes it plays an important role in fatty acid and polyketide biosynthetic pathways. Moreover, this enzyme generates the substrate that is used in the acetylation of protein lysine residues. In a wide variety of organisms ACS activity is known to be regulated by reversible post-translational acetylation. Specifically, the ACS from *Mycobacterium tuberculosis* can be auto-acetylated (Li et al., 2011). In addition, the enzymatic acetylation of a lysine residue, as a mechanism of ACS regulation, has also been studied in bacterial systems (Gardner et al., 2006; Bernal et al., 2014; Noy et al., 2014). For example, the non-acetylated ACS of *Salmonella enterica* is about 480-fold more active than the acetylated enzyme (Starai et al., 2002). The deacetylation of this enzyme is catalyzed by CobB, which is a homolog of the yeast Sir2 deacetylase (Tsang and Escalante-Semerena, 1998). The acetylation of ACS occurs at the side chain of a Lys residue that is positioned in the middle of a conserved structural motif, called A10 (Gulick, 2009). Structural studies of the *S. enterica* ACS indicate that this acetylation does not induce any conformational changes in the enzyme (Gulick et al., 2003). However, acetylation significantly affects the ACS first half-reaction, presumably by aligning the acetate-substrate with the active site residue(s) (Gulick et al., 2003); this protein acetylation reaction does not affect the second half-reaction catalyzed by *S. enterica* ACS (Starai et al., 2002).

In plant systems, such as Arabidopsis, the metabolic function of ACS is not completely clear (Fu et al., 2020). Despite the fact that ACS is plastid localized (Lin and Oliver, 2008), unlike other biological systems, ACS is not the physiological source of acetyl-CoA that is the precursor of the plastid-localized *de novo* fatty acid biosynthesis process (Ke et al., 2000). Rather, it appears that ACS in plants is important in maintaining acetate homeostasis; acetate being toxic at higher levels of accumulation (Fu et al., 2020). In this study, we explored if the acetylation-based regulation of ACS is extrapolatable from bacteria, yeast and algae to affect the activity of this enzyme in plant systems. Specifically, this was explored by a combination of site-specific mutagenesis strategies, including a strategy for expanding the genetic code, and thereby *de novo* reconstruct the post-translational modification of this protein (Chen et al., 2018a). The advantage offered by this latter strategy is the ability to generate homogeneous preparations of modified proteins, and thus is a powerful technique to study post-translational modifications (Johnson et al., 2010). In this study, we used this strategy to explore the post-translational acetylation of an ε-amine lysine group of the Arabidopsis acetyl-CoA synthetase (atACS) as a potential regulatory mechanism.

## 2 Materials and methods

### 2.1 Generation of atACS variants

The ORF sequence coding for the mature atACS that lacks the chloroplast targeting sequence has been extensively characterized in previous studies (Ke et al., 2000; Behal et al., 2002; Lin and Oliver, 2008; Sofeo et al., 2019). Using this pET30f-based plasmid as the template, we used QuikChange Lightning Site Mutagenesis Kit (Agilent Technologies, Santa Clara, CA) to generate point mutants at the Lys-622 codon, i.e., the Lys622Ala, Lys622Arg and Lys622Gln variants. To generate the Lys622AcK variant, the wild-type atACS ORF sequence was cloned into the pCDF-1b vector, which genetically fused a Hexa-His-tag at the C-terminus of the atACS sequence. Subsequently, the codon encoding for lysine-622 was mutated to the “TAG” termination codon. All point mutant variants were confirmed by direct sequencing of the plasmid products. The plasmid carry the atACS Lys622TERM codon variant was co-transformed with the *pTech*-based plasmid, which expressed the orthogonal tRNA and an engineered pyrrolysyl-tRNA synthetase variant (Venkat et al., 2017a; Venkat et al., 2017b; Venkat et al., 2018) (a kind gift obtained from Dr. Chenguang Fan, Department of Chemistry & Biochemistry, University of Arkansas) into an *E. coli* strain and propagated in media containing streptomycin (50 μg/mL) and chloramphenicol (50 μg/mL) to maintain both plasmids.

### 2.2 Expression and purification of wild-type and variant proteins

Wild-type atACS and all the lysine-622 mutant variants were expressed in either *E. coli* BL21(DE3) or Arctic Express(DE3) strains, and purified as earlier described (Sofeo et al., 2019). In brief, the strains carrying an expression plasmid vector was grown overnight at 37°C with agitation at 250 rpm in a Innova 4300 incubator shaker (New Brunswick Scientific, Edison, NJ), in 5–10 mL LB medium containing the appropriate antibiotics. The overnight culture was used to inoculate 0.25 L LB media containing the same antibiotics. These cultures were grown at 37°C with agitation at 250 rpm, until the OD_600_ reached ∼0.6. Protein expression was then induced by the addition of IPTG to a final concentration of 0.4 mM, and cultures were incubated with agitation at 250 rpm for 24–48 h, at either 22°C [for BL21(DE3) strain] or 13°C [for Arctic Express(DE3) strain]. For the expression of the AcK atACS variant, this final culture medium also contained 20 mM nicotinamide to inhibit the endogenous cobB deacetylase (Venkat et al., 2017a; Gallego-Jara et al., 2017), and 2 mM N-ε-acetyl-lysine (Sigma-Aldrich Co., St. Louis, MO), to ensure that this non-canonical amino acid did not limit the expression of the AcK-variant.

Wild-type and variant atACS proteins were purified by a process that we had previously developed (Sofeo et al., 2019). For the purification of the AcK atACS variant, all buffers contained 20 mM nicotinamide to inhibit cobB deacetylase that maybe contaminating the preparations. The purification procedure was initiated by harvesting *E. coli* cells by centrifugation at 10,000 g for 10 min. The pellet was resuspended in a buffer containing 0.5 M NaCl, 5 mM imidazole, 0.1% (v/v) Triton X-100, 0.1 mg/mL phenyl methyl sulfonyl fluoride and 10 μL/mL Protease Inhibitor Cocktail (Sigma-Aldrich Co., St. Louis, MO). The cell pellet was disrupted by sonication on ice and subsequently the suspension was centrifuged at 20,000 g for 30 min. The supernatant was filtered through a 0.45 μm filter disc (Corning Inc. Corning, NY) and applied to a column containing 2 mL PerfectPro Ni-NTA agarose (5 Prime, Inc. Gaithersburg, MD) at 4°C. The column was washed with increasing concentrations of imidazole, and the atACS protein was eluted with 0.2 M imidazole. The purified proteins were immediately dialyzed into 10 mM HEPES-KOH, pH 7.5, 10 mM KCl, 2 mM TCEP, 10% glycerol, concentrated and either characterized immediately or flash frozen in liquid nitrogen and stored at −80°C.

### 2.3 Gel filtration chromatography

Purified atACS proteins were subjected to size exclusion gel filtration chromatography using an AKTA FPLC system (GE Healthcare Life Sciences, Pittsburg, PA) with a Superdex 200 Increase 10/300 GL gel filtration column (GE Healthcare Life Sciences, Pittsburg, PA). A 100 µL aliquot of purified atACS protein (4–10 mg/mL) was injected into the prepacked column, and chromatography was conducted with a buffer consisting of 10 mM HEPES-KOH, pH 7.5, 10 mM KCl, 2 mM TCEP, 10% glycerol, eluted at a rate of 0.4 mL/min; the eluate was monitored using a UV absorbance detector, at 280 nm.

### 2.4 Autoacetylation of isolated atACS

The autoacetylation of atACS (∼130 µg of purified protein) was conducted in a 1-mL volume of a buffer consisting of 10 mM potassium acetate, 10 mM MgCl_2_, 10 mM ATP, 50 mM Tris-HCl, pH 8.0 (Li et al., 2011). Following incubation at 37°C for 2 h, the protein solution was dialyzed into 10 mM HEPES, pH 7.5, 10 mM KCl, 2 mM TCEP, 10% glycerol.

### 2.5 Protein analysis

Protein preparations were evaluated by SDS-PAGE, and gels were either stained with Coomassie Briliant Blue, or the acetylation status of atACS was evaluated by Western blot analysis, using an antibody (1:1,000 diluted) that reacts with *N*-ε-acetyl-L-lysine (Cell Signaling Technology, #9441). The acetylation status of atACS was also evaluated by mass spectrometric analysis of rLysC (Promega Corporation, Madison, WI) digested protein, using Q Exactive™ Hybrid Quadrupole-Orbitrap Mass Spectrometer (Thermo Fisher Scientific Inc. Waltham, MA), housed at the Iowa State University Protein Facility (http://www.protein.iastate.edu). The purified protein preparations of wild-type and Ac-K atACS variants were subjected to SDS-PAGE analysis. Following staining with Coomassie Brilliant Blue, the protein band of interest was excised and digested using an Investigator™ ProGest (Genomic Solutions, Digilab Inc., Hopkinton, MA) in 0.5 mL buffer (50 mM Tris, pH 8, 15 mM iodoacetamide and 5 mM DTT) containing 20 µg LysC per mg of purified atACS protein. The peptides were identified using Sequest-HT (Eng et al., 1994) as the search engine within Proteome Discoverer (PD) 2.2 (Version 2.2.0.388; Thermo Fisher Scientific). The peptides were analyzed against the ACS protein sequence with settings for four possible missed cleavage sites, with fragment mass tolerance of 0.02 Da precursor mass tolerance of 10 ppm. The possible side-chain modifications that were analyzed include the dynamic acetylation of Lys, static carbamidomethylation of Cys, dynamic deamidation of Asn and Gln and dynamic oxidation of Met residues. The purified intact atACS proteins (wild-type and Lys-622 variants) were also analyzed by mass spectrometry using a Waters SYNAPT G2-Si High Definition Mass Spectrometer, at the Iowa State University Protein Facility to determine the molecular weight of each protein. The purified protein preparations were dialyzed in water to remove salts, buffer and glycerol contaminants, prior to analysis.

### 2.6 Spectrophotometric atACS activity assay

AtACS activity was measured by coupling the acetate- and CoA-dependent formation of AMP from ATP, to the oxidation of NADH, using the reactions catalyzed by myokinase, lactate dehydrogenase and pyruvate kinase (Sofeo et al., 2019). In brief, assays were performed at 37°C in a final volume of 100 μL, in individual wells of 96-well microtiter dishes. The absorbance of NADH was measured at 340 nm using a BioTek ELx808TM Absorbance Microplate Reader, and data were collected and analyzed with Gen5TM Data Analysis software (BioTek Instruments, Winooski, VT). The reaction mix initially contained 50 mM Tris-HCl pH 7.5, 5% (v/v) ethanol, 5 mM Tris(2-carboxylethyl) phosphine hydrochloride (Thermo Fisher Scientific Inc. Waltham, MA), 6 mM MgCl_2_, 5 mM ATP, 5 mM phospho(enol)pyruvate, 0.4 mM NADH, 2–20 U Pyruvate Kinase/Lactate Dehydrogenase mix (Sigma-Aldrich Co., St. Louis, MO), 1U Myokinase from chicken muscle (Sigma-Aldrich Co., St. Louis, MO) and 5 μg of recombinant atACS protein. After monitoring the initial background change A_340_, ACS activity was initiated by the addition of 2.5 mM CoA, and progress of this reaction was monitored as a decrease in A_340_. All data were collected from three independent assays, and all raw data collected for this study are provided in Supplementary Table S1.

### 2.7 Circular dichroism spectroscopy

CD spectra were obtained with a Jasco J-710 CD spectrometer (JASCO Analytical Instruments, Easton, MD) at the Iowa State University Chemical Instrumentation Facility (https://www.cif.iastate.edu). Spectra were taken at wavelength range of between 190 nm and 260 nm, with the following settings parameter: pitch point 1, speed 20 nm/min, response time 4 s, bandwidth 1 nm, temperature 20°C and data mode CD-HT. Spectra where acquired with a 1 mm dichroically neutral quartz cuvette and the average of 3 scans was used for analysis. Acquisition of spectra were obtained with protein samples in 10 mM HEPES-KOH, pH 7.5, 10 mM KCl, 2 mM TCEP and 10% glycerol, and these solutions were diluted into double distilled H_2_O to a final protein concentration of 0.1–0.2 mg/ml. A baseline-spectrum for the same dilution of the buffer was obtained and subtracted from the experimental protein spectrum. Collected spectral data were converted from millidegrees to molar ellipticity ([θ], degrees cm^2^.dmol^−2^). Spectra were analyzed using a suite of algorithms collectively called CDPro (Sreerama and Woody, 2000), utilizing algorithms: SELCON3, CDSSTR, and CONTIN/LL.

## 3 Results

**FIGURE 1:**
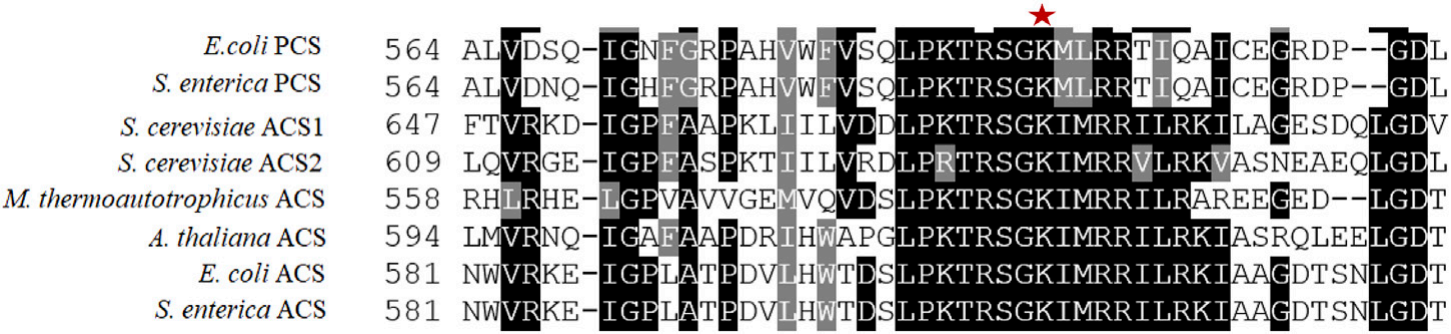
Comparison of the amino acid sequences of motif A10 of the AMP-forming family of acyl-CoA synthetases. Sequence alignments of enzymes that use acetate (ACS) or propionate (PCS) as substrates from different organisms. The red asterisk identifies the conserved lysine residue that is reversibly acetylated. Alignment was performed with Clustal Omega, and white letters on a black background indicate identity, white letters on a gray background indicate similarity, and black letters on a white background indicate no conservation.

The atACS sequence shares considerable similarity with ACS sequences from a range of diverse biological phyla (Sofeo et al., 2019), and these occur in 10 conserved motifs (A1 to A10) that are distributed evenly throughout the length of these sequences (Gulick, 2009). Three of these motifs (A3, A4, and A5) contain conserved residues that determine the carboxylate substrate specificity of this class of enzymes (Marahiel et al., 1997; Gulick et al., 2003; Starai and Escalante-Semerena, 2004). Motif A10 contains a highly conserved lysine residue (at position 622 of the Arabidopsis enzyme) (Figure 1). Based on studies of homologous proteins, this lysine residue of the Arabidopsis enzyme may be a target for reversible acetylation, a mechanism that regulates the catalytic capability of these enzymes (Starai et al., 2002).

**FIGURE 2:**
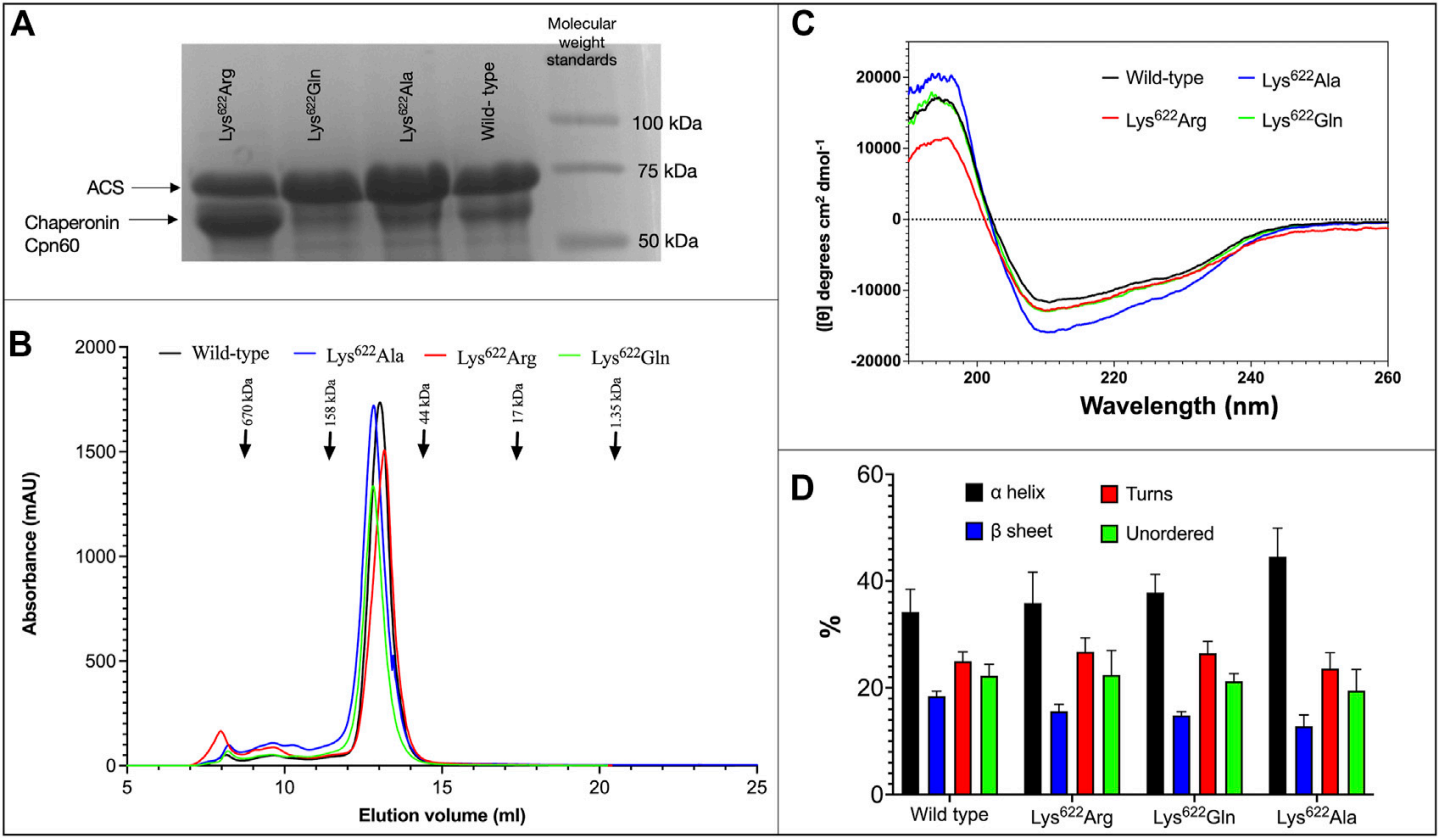
Characterization of ACS Lys622 mutants. **(A)** SDS-PAGE analysis of purified wild-type and indicated Lys622 mutant variants of atACS (see Supplementary Figure S6 for whole gel image). **(B)** Size exclusion gel filtration chromatography of wild-type and Lys622 mutant variants of atACS. **(C)** CD spectra of wild-type and Lys622 mutant variants of atACS. Measurements are an average of three replicate scans. **(D)**. Secondary structure composition calculated from the CD spectra of wild-type and Lys622 mutant variants of atACS. Errors bars indicate standard errors from 3 replicate CD spectra calculated by 3 algorithms, SELCON3, CDSSTR, and CONTIN/LL.

Several different experimental strategies were used to evaluate whether acetylation of residue Lys^622^ of atACS modulates catalytic activity of the enzyme. One of these experiments focused on the characterization of mutant variants, in which the Lys^622^ residue was mutated to Ala, Gln or Arg residues. These variant proteins behaved similarly to each other and to the wild-type enzyme in such attributes as: a) stability and yield upon expression in *E. coli*, recovering 10–15 mg of purified protein/L of culture (Figure 2A); b) elution from size exclusion gel filtration chromatography occurs as a single symmetric peak, at an elution volume consistent with the molecular weight of a ∼72-kDa monomer (Figure 2B); c) CD spectra show that all variant proteins are similarly folded as the wild-type enzyme, and secondary structure calculations indicate insignificant differences in the proportion of α-helices, β-sheet, turns and unordered secondary structures among the 3 variant proteins as compared to the wild-type protein (Figures 2C, D).

**FIGURE 3:**
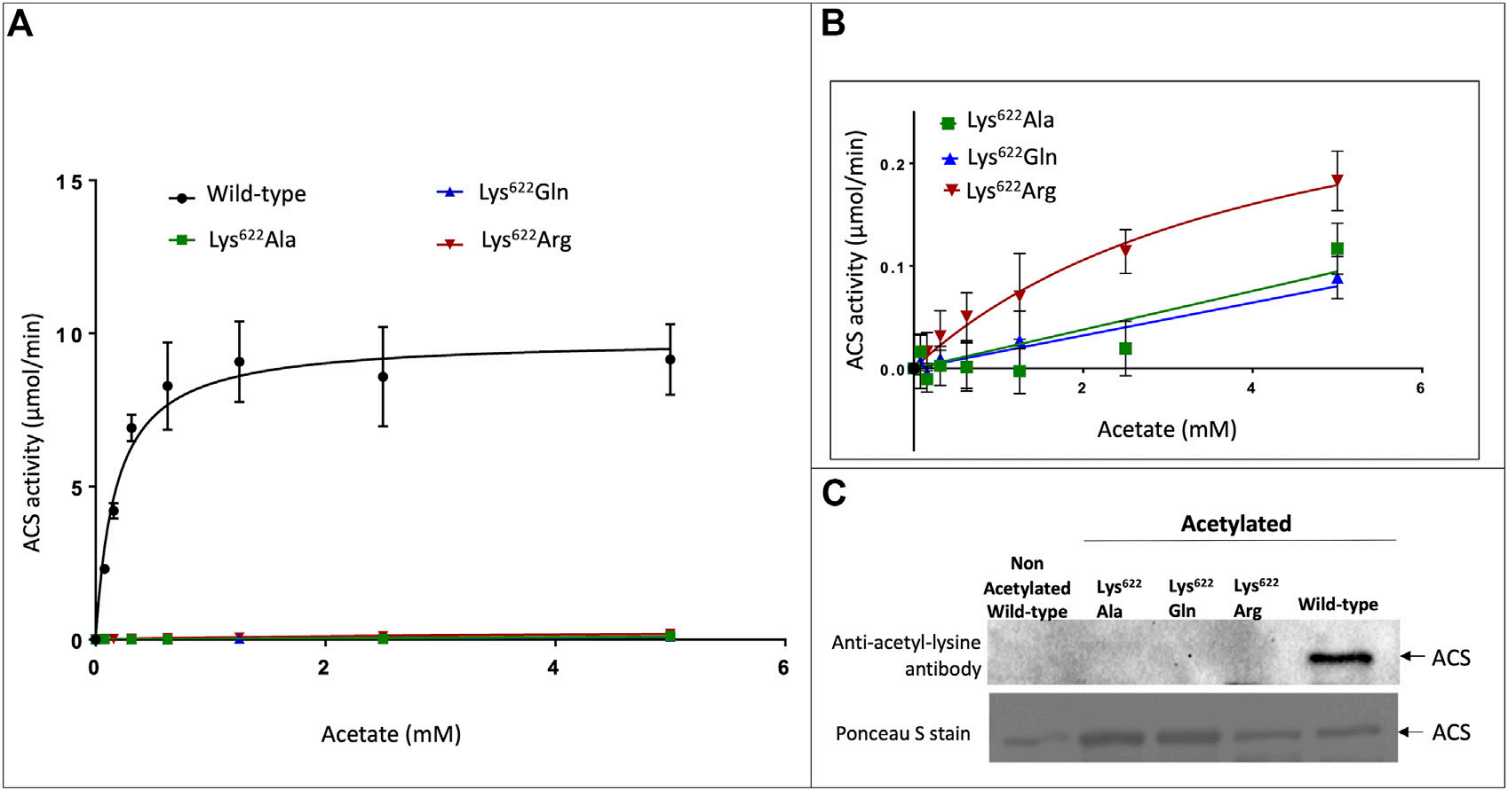
Effect of acetylation on catalytic properties of atACS. **(A)** Michaelis-Menten kinetic analysis of atACS Lys622 variants. **(B)** Expanded view of data presented in Panel (A). **(C)** Acetylation status of atACS Lys622 variants, analyzed by Western blot analysis with anti-acetyl-lysine antibody. The top panel is the result from Western blot analysis and bottom panel shows Ponceau S staining of the same membrane (see Supplementary Figure S6 for whole gel image). Data-points in panels A and B are the average of triplicate independent determinations, and error bars represent the standard deviation.

Enzymatic assays of the three Lys^622^ variants demonstrate that compared to the wild-type enzyme, catalytic competence of these variants is reduced by more than 30-fold. Specifically, the k_cat_ for the Lys^622^Arg mutant is reduced by 30-fold (increasing K_m_ by 25-fold), while the catalytic activity of the Lys^622^Ala and Lys^622^Gln mutants is even further reduced to such low levels that catalytic constants could not be accurately determined (Figures 3A, B). The almost complete loss of catalytic competence by the latter two mutants is consistent with the critical role of Lys^622^ in the catalytic mechanism of this enzyme. Specifically, the positive charge of the ε-amine group of this lysine residue stabilizes the developing negative charge on the α-P atom of ATP during the first half reaction that forms the acetyl-adenylate intermediate (Gulick et al., 2003; Jogl and Tong, 2004; Gulick, 2009; Noy et al., 2014). The inability of Arg to replace the functionality of the Lys^622^ residue is probably due to the larger size of the Arg side-chain, which would not enable the latter to be an exact phenocopy of the deacetylated protein, and this has previously been reported for homologous ACS enzymes from bacteria and mammals (Dancy and Cole, 2015; Wang et al., 2017).

Gel filtration FPLC analysis of the purified wild-type and Lys^622^ mutant variants of atACS were chemically autoacetylated *in vitro*, and their acetylation status was determined by Western blot analysis using the anti-acetyl-lysine antibody. While the wild-type enzyme is acetylated, as expected all the Lys^622^ variants are not acetylated (Figure 3C). Therefore, these data indicate that this residue may be the site of acetylation of the atACS, which affects enzymatic competence of this enzyme.

Direct assessment of this hypothesis was evaluated by incorporating acetyl-lysine (AcK) specifically at position #622 of atACS. This was accomplished by first engineering a termination codon into the ORF sequence at position-622. The expression of this variant in the absence of AcK results in the expression of a truncated atACS protein that terminates at codon #622. However, when AcK is provided in the medium, because of the expression of a cognate tRNA to read the termination codon as acetyl-lysine (Venkat et al., 2017a; Venkat et al., 2018), AcK is incorporated at position 622, and the resultant atACS-AcK variant is translationally fused at the C-terminus to a His_6_-tag that is part of the expression vector used in these studies. Thus, Ni-column affinity chromatography was used to purify this atACS-AcK-His_6_-tag variant. SDS-PAGE analysis of the recovered protein preparations identified two proteins bands, close to the expected 72-kDa band of atACS (Supplementary Figure S2A). Size exclusion gel filtration chromatography of the protein preparations identified two UV-absorbing peaks (A and B) (Supplementary Figure S2B). The fractions that encompass these the two protein peaks were pooled separately as indicated (Supplementary Figure S2B) and concentrated for further analysis.

These two protein-preparations were subjected to chymotrypsin digestion, and resulting peptides were analyzed by mass-spectrometry to determine their identity. The Peak B derived peptide sequences showed 55% coverage of the atACS sequence, whereas Peak A peptides showed only 4.5% coverage of the atACS sequence (Supplementary Figures S3, S4). Additionally, enzymatic assays of Peak A and B preparations indicate that Peak A does not support ACS catalytic activity, whereas Peak B supports the catalysis of acetyl-CoA synthesis (Supplementary Figure S5). Based on these results therefore, we conclude that Peak A represents a contaminating protein, and that Peak B is the atACS-AcK variant, and all further characterizations were conducted by pooling fractions that encompassed Peak B.

**FIGURE 4:**
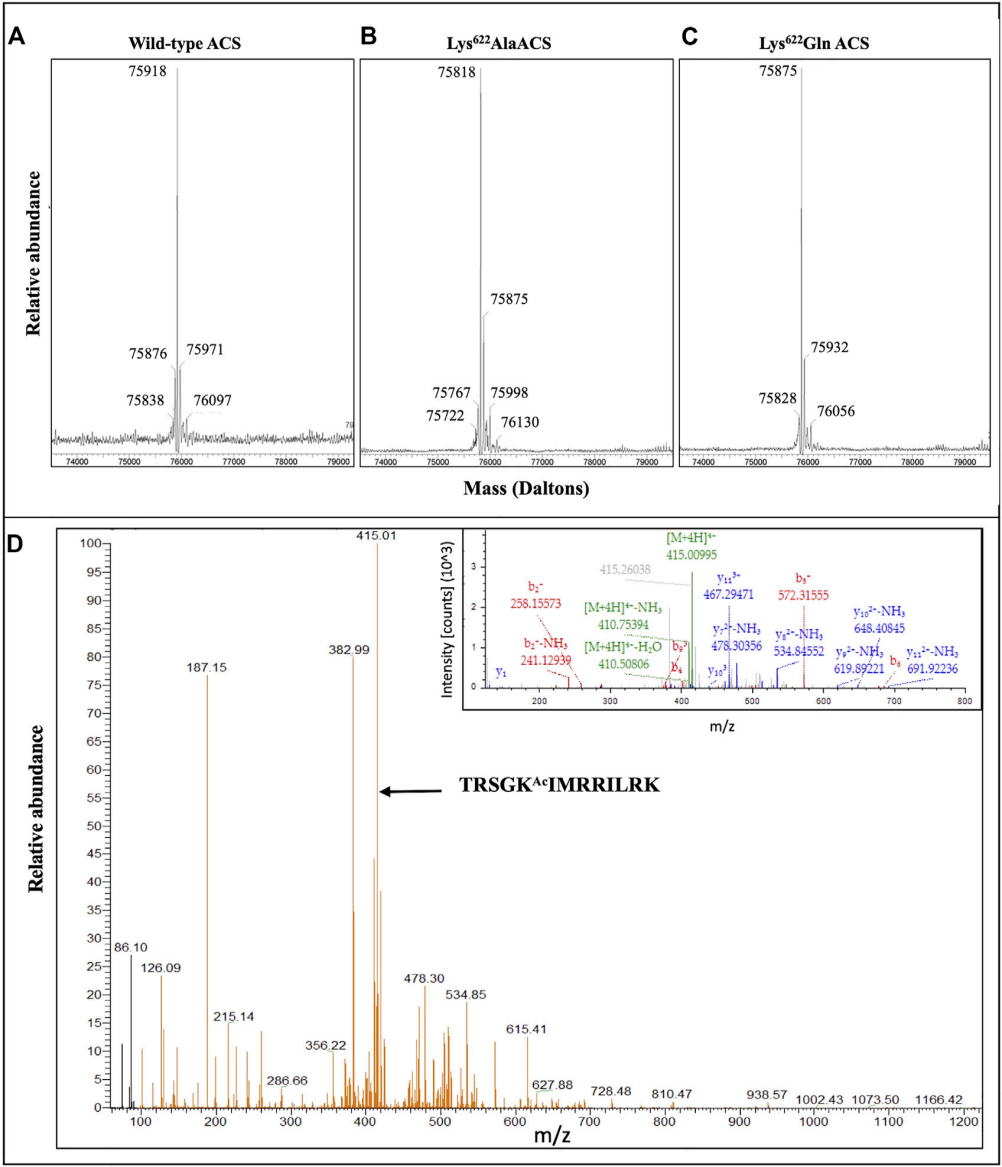
Mass spectrometric analyses of acetylated atACS. Purified wild-type **(A)**, and Lys622Ala **(B)** and Lys622Gln **(C)** variants of atACS were *in vitro* autoacetylated and their molecular weights determined by mass spectrometry with a Waters SYNAPT G2-Si High Definition Mass Spectrometer. The theoretical molecular weights of these unmodified proteins are predicted to be 75,875.97 Da, 75,818.87 Da and 75,875.93 Da, respectively. **(D)** Mass spectrometric identification of acetylated Lys-622 containing peptide isolated from purified atACS AcK variant. LC-MS/MS spectra and MS2 fragmentation pattern (inset) of the LysC generated peptide containing lysine 622 from atACS Ac-K variant.

The purified mature His-tagged atACS proteins were characterized by mass spectrometry. These analyses focused on auto-acetylated wild-type atACS (Figure 4A), and auto-acetylated Lys^622^Ala atACS and Lys^622^Gln atACS mutants (Figures 4B, C). Based upon the expected amino acid sequence, the theoretical molecular mass of the unmodified wild-type His-tagged atACS protein is predicted to be 75875.97 Da. This compares with the experimentally determined mass of the acetylated His-tagged atASC protein, of 75918 Da (Figure 4A), a difference of 43 Da, consistent with a single acetylation event. Examination of this mass spectrum indicates the presence of both the acetylated (the 75918 Da ion) and non-acetylated (the 75876 Da ion) forms of atACS, with the former ion being ∼10-fold more abundant than the latter. Moreover, because the experimentally determined masses of the Lys^622^Ala atACS and Lys^622^Gln atACS mutants treated for auto-acetylation (Figures 4B, C) are in agreement with the predicted molecular masses of the unmodified proteins (i.e., 75818.87 and 75875.93), we conclude that acetylation of atACS occurs only at Lys^622^, and that there are no other sites of modification.

Finally, direct demonstration that the isolated atACS-AcK variant is acetylated at the targeted Lys^622^ residue was obtained by mass-spectrometric analysis of the LysC-digested protein, and these data were compared to the mass-spectrometric analysis of the LysC digested wild-type atACS protein. The sequence coverage from these analyses were 40% for the wild-type atACS protein, whereas coverage of the atACS-Ack variant was ∼62% (data not shown). Although the lysine-622 containing peptide could not be identified in the LysC digest of the wild-type atACS, it was successfully detected from the atACS-Ack variant protein. The acetylated lysine-622 peptide sequence was identified as, TRSGK^Ac^IMRRILRK. This was based on the monoisotopic m/z value of 415.01038 Da (+0.76 mmu/+1.84 ppm), with a charge of +4 and theoretical mass of the MH + ion of 1657.01967 Da (Figure 4D).

**FIGURE 5:**
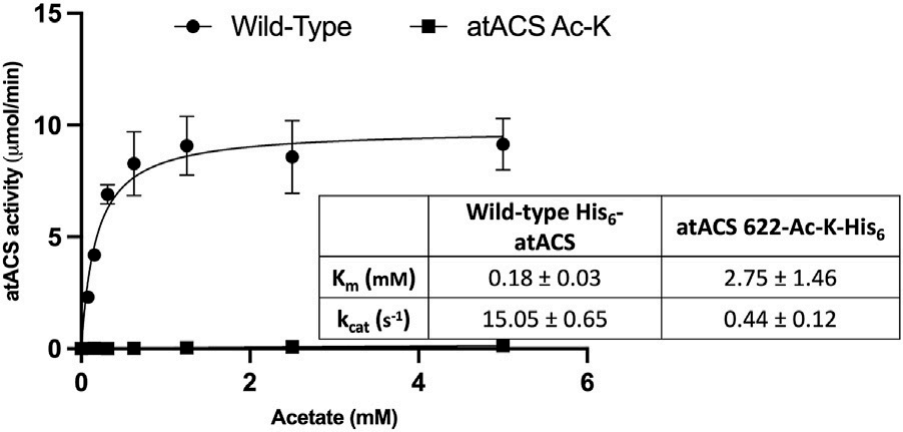
Catalytic capabilities of wild-type atACS and atACS Ac-K variant. The inset table shows the Michaelis-Menten kinetic parameters of His-tagged wild-type atACS and atACS Ac-K variant. Data-points are the average of triplicate independent determinations, and error bars represent the standard deviation.

Comparing the Michaelis-Menten kinetic parameters (K_m_ and k_cat_) of atACS to the atACS-Ack variant indicates that acetylation of the enzyme affects its ability to catalyze acetyl-CoA synthesis. Specifically, as compared to the wild-type atACS, the atACS-Ack variant shows a 34-fold decrease in k_cat_, and a 15-fold increase in K_m_ for the acetate substrate (Figure 5).

## 4 Discussion

It has become increasingly apparent that the post-translational acetylation-deacetylation cycle of the ε-amino group of lysine residues is an important mechanism for regulating many biological processes (Dancy and Cole, 2015; Chen et al., 2018a; Christensen et al., 2019; Hossain and Tsang, 2019; Hu et al., 2019; Wang and Wang, 2019). This modification reaction is one of the broader suites of mechanisms by which the chemo-physical properties of side chains of amino acid residues can be diversified from the 20 genetically encoded canonical amino acids. Moreover, because these modifications are often reversible, and enzyme catalyzed, these reactions provide a rapid mechanism for regulating biological processes. Specifically, one can rationalize that acetylation of the ε-amino group of a lysine residue changes the character of that side chain from a highly polar, positively charged residue to a non-polar side chain that is capable of participating in weaker H-bonding interactions as both a H-bond donor or H-bond acceptor. Such drastic changes in the properties of such a side chain can generate molecular forces that confer structural alterations in proteins, which thereby render altered functionality.

Because ACS has a role in generating the substrate for such acetylation modification reactions, the post-translational acetylation-deacetylation of this enzyme has potential to generate an autoregulatory loop that could enhance or dampen such controlling mechanisms. Indeed, in microbes and mammals ACS catalytic activity can be regulated by the acetylation of a specific lysine residue that occurs in a conserved region of these enzymes (Starai et al., 2003; Gardner et al., 2006; Hallows et al., 2006; Schwer et al., 2006; Noy et al., 2014). For example, acetylation of a specific lysine residue in the A10 motif of the bacterial ACS inactivates catalysis (Gardner et al., 2006; Noy et al., 2014). Similarly, with the mammalian enzyme, acetylation completely deactivates catalysis, and deacetylation by sirtuin reactivates enzymatic activity (Hallows et al., 2006; Schwer et al., 2006). In contrast, acetylation of the analogous lysine residue in homologous long chain acyl-CoA synthetases increases catalytic activity (Chen et al., 2018b).

Moreover, global proteomics studies have identified that the acetylation of this specific Lys residue occurs *in vivo* in many bacteria, fungi and animals (Gulick et al., 2003; Desiere et al., 2006), including the well characterized *E. coli* and *S. enterica* ACS. Unfortunately, the Lys-622 residue that was the subject of this study is in a region of the atACS protein that contains a plethora of Lys and Arg residues, which has prevented it from being detected in global proteomics studies that typically utilize trypsin as the protease to generate peptides for MS analysis. In this study therefore, a series of experiments were conducted that expanded upon prior studies and demonstrated by a combination of different strategies that the plastid localized plant ACS is susceptible to post-translational acetylation mechanism, which can modulate catalytic capability. Specifically, our Western blot and mass spectrometric studies demonstrate that acetylation of Lys-622 of atACS reduces catalytic efficiency (k_cat_/K_m_) of the enzyme by ∼522-fold. The importance of Lys-622 acetylation to ACS catalysis was demonstrated by a number of mutagenesis experiments. Specifically, eliminating this side chain, as in the Lys622Ala variant eliminates catalysis. Moreover, the conservative substitution of this residue (i.e., Lys622Arg variant), which maintains the positive side chain characteristic also eliminates ACS catalysis. Furthermore, the substitution of Lys-622 with a residue that is capable of H-bond formation (i.e., Lys622Gln variant) has the same inactivation result. These mutations do not affect the overall gross folding, oligomeric state, or the *in vivo* stability of the recombinant atACS protein, even though it renders the enzyme inactive. Hence, these results indicate the importance of this lysine residue in supporting the catalytic mechanism of this enzyme, but do not directly address whether this Lys residue is acetylated.

We therefore used an alternative strategy to address this question, namely, using an already acetylated lysine residue that was genetically inserted into the protein sequence at the target position during the *in vivo* synthesis of the atACS protein. Such a strategy has been used to not only incorporate N-ε-acetyl-lysine at targeted sites, but also a number of different non-canonical amino acids (Johnson et al., 2010). The strategy uses an orthogonal pair of a suppressor tRNA and its respective amino acyl tRNA synthetase that recognizes a specific non-canonical amino acid, and that amino acid is genetically programmed to be incorporated during the translation process of a host, typically *Escherichia coli* (Chen et al., 2018a). The position for incorporating the non-canonical amino acid is defined by termination-codon mutagenesis of the protein-coding ORF, which is recognized by the anticodon of the suppressor tRNA that carries the non-canonical amino acid (Xie and Schultz, 2006). Using this strategy we generated homogeneous preparations of modified protein (i.e., atACS-Ack) for characterization. Comparing the kinetic parameters of the wild-type and atACS-Ack variant, we found that the acetylated enzyme showed an increase in the K_m_ and decrease in the k_cat_ values for the acetate substrate. Collectively therefore, these results establish that acetylation of Lys-622 of atACS results in the inactivation of its catalytic capability. Hence, reversible acetylation of this residue would be an important mechanism by which the activity of this enzyme is modulated *in vivo*, especially to accommodate changes in cellular acetate and/or acetyl-CoA levels.

Consistent with its role in catalysis, the experimentally determined structure of the acetylated and non-acetylated bacterial ACS (Gulick et al., 2003; Fox et al., 2017) has established that this Lys residue is near the active site pocket during the first half of the reaction, when acetate and ATP bind and react to form the acetyl-adenylate intermediate and release the pyrophosphate product (Gulick et al., 2003; Gulick, 2009). MStructural modeling of the atACS indicates a similar configuration for Lys-622, which indicates that acetylation inhibits the first half-reaction catalyzed by atACS.

The *in vivo* physiological role of ACS in plants was initially thought to be the enzymatic supplier of the acetyl-CoA substrate required for *de novo* fatty acid biosynthesis in plastids (Behal et al., 2002). However, genetic and biochemical characterizations established that ACS is not needed for this metabolic role (Ke et al., 2000), and more recent genetic characterizations establish that ACS is part of a 2-enzyme system that plants use to maintain acetate homeostasis (Fu et al., 2020). Because acetyl-CoA is a crucial intermediate of metabolism that juxtaposes anabolic and catabolic processes, and is also a critical component of many regulatory processes associated with acetylation of controlling components (e.g., histone acetylation or N-terminal and/or amino acid side-chain acetylation), one can envision that its generation is highly regulated. Hence, the post-translational acetylation mechanism identified herein for modulating atACS activity could provide plastids of plants cells with the ability to rapidly adapt to changing environmental and developmental conditions and maintain cellular acetyl-CoA and acetate homeostasis.

## Data Availability

The original contributions presented in the study are included in the article/Supplementary Material, further inquiries can be directed to the corresponding author.

## Supplementary material

Supplementary Figure S1 Purification of atACS-AcK variant. **(A)**. Coomassie stained SDS-PAGE analysis of the purified protein preparations. Wild-type atACS and atACS-AcK carried a His6-tag at N-terminus and C-terminus, respectively. **(B)**. Size exclusion gel filtration chromatography of three preparations of atACS-Ack variants. Aliquots of each peak; Peak A (fractions A7-A10) and Peak B (fractions B10-B7), were analyzed by SDS-PAGE and stained with Coomassie Brilliant Blue (shown under the elution profile). The indicated fractions were pooled and subjected to further analysis (see Supplementary Figure S5 for whole gel image).

Supplementary Figure S2 Green shaded residues pinpoint the chymotryptic-digest peptides identified by mass spectrometric analysis of the Peak A protein (Supplementary Figure S1), mapped to the atACS sequence; coverage is 4.5% of the atACS sequence.

Supplementary Figure S3 Green shaded residues pinpoint the chymotryptic-digest peptides identified by mass spectrometric analysis of the Peak B protein (Supplementary Figure S1), mapped to the atACS sequence; coverage is 55% of the atACS sequence.

Supplementary Figure S4 Acetate dependence of the ACS activity of the FPLC purified atACS-AcK variant (Peak B). Peak A did not show any detectable activity.

Supplementary Figure S5 Whole gel images from Figures Figure 2A, Figure 3C, and Supplementary Figure S1.

Supplementary Table S1 Raw data of activity assays of wild-type and variant atACS enzymes.
