# Supplementary figures and images for: Modulation of plant acetyl-CoA synthetase activity by post-translational lysine acetylation
Source: Front Mol Biosci. 2023 Mar 16;10:1117921. doi: 10.3389/fmolb.2023.1117921 (PMC10062202; doi:10.3389/fmolb.2023.1117921)

**Supplemental Figure S4**

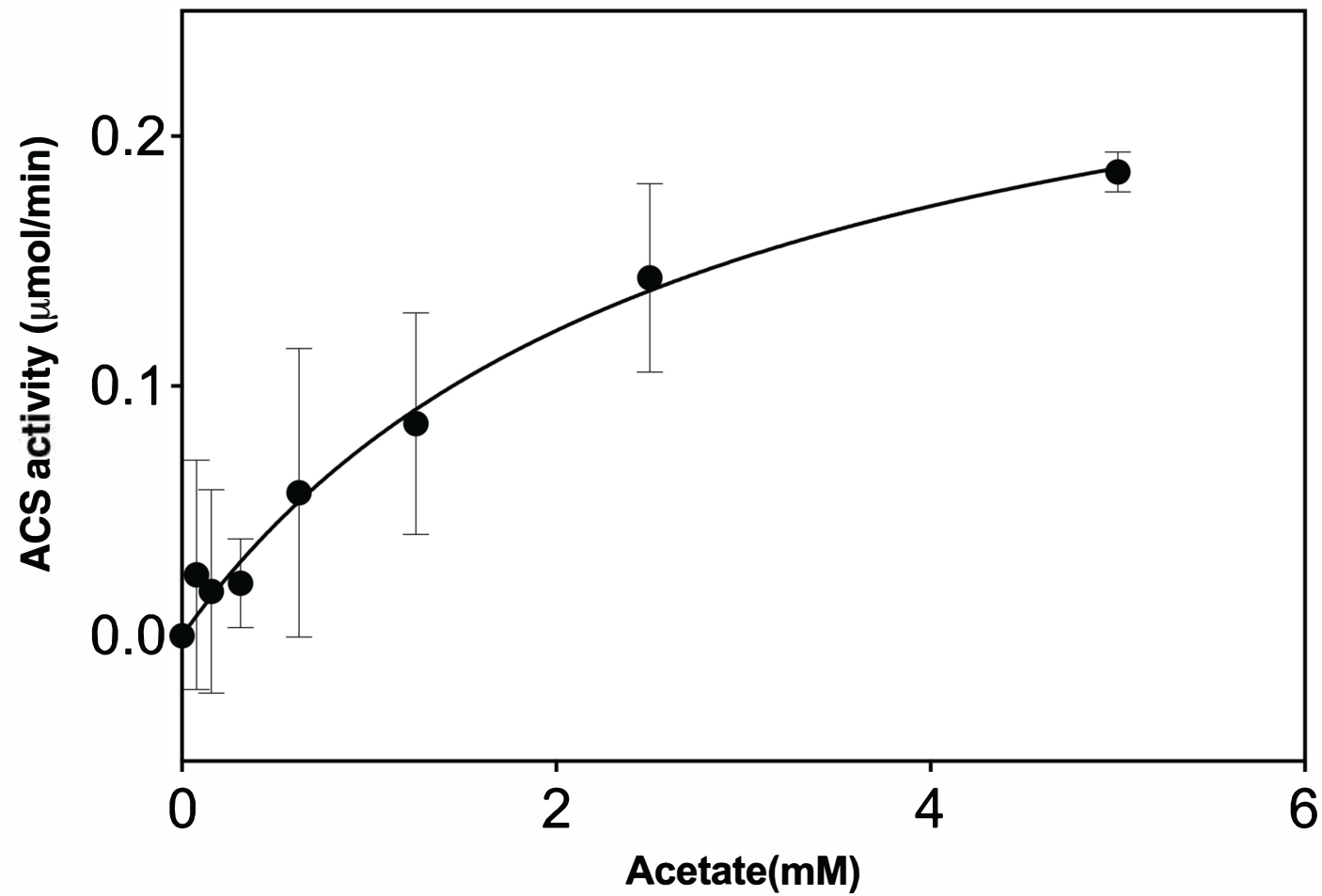

Supplement: Supplementary file 2 [file Image4.pdf]

Supplemental Figure S1

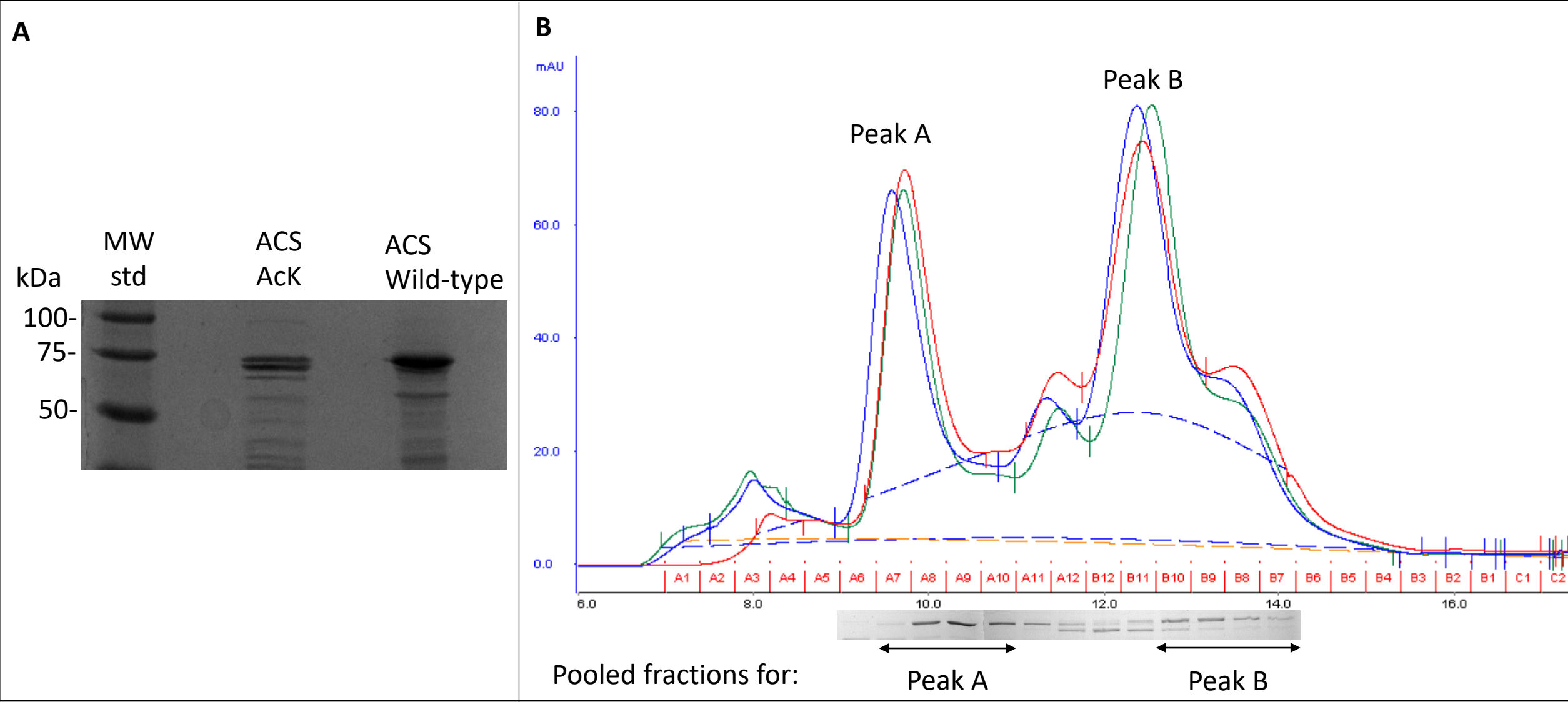

Supplement: Supplementary file 6 [file Image1.pdf]
