# Supplementary material for: Modulation of plant acetyl-CoA synthetase activity by post-translational lysine acetylation
Source: Front Mol Biosci. 2023 Mar 16;10:1117921. doi: 10.3389/fmolb.2023.1117921 (PMC10062202; doi:10.3389/fmolb.2023.1117921)
Supplement: Supplementary file 3 [file Image2.pdf]

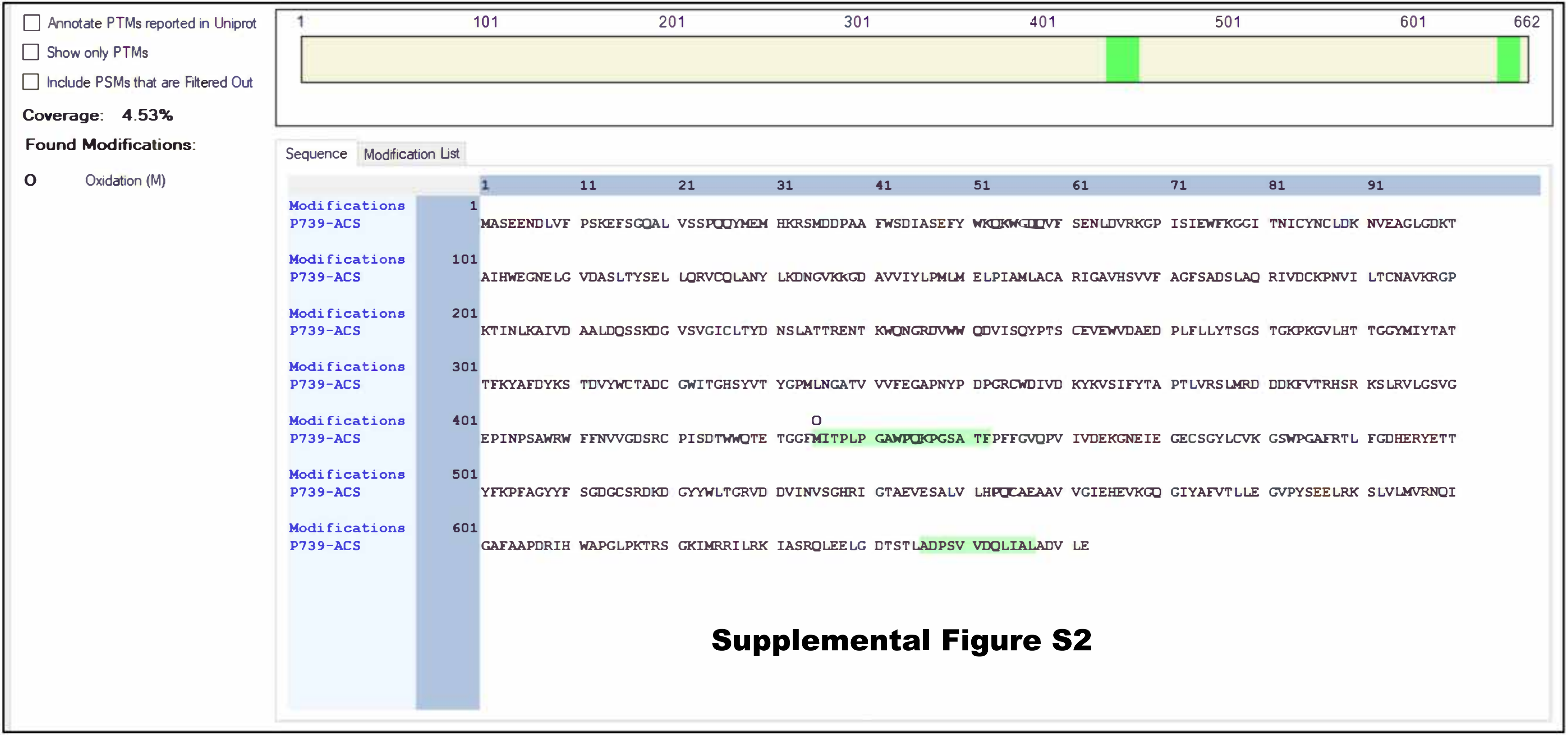

Supplemental Figure S2
