# Supplementary material for: Modulation of plant acetyl-CoA synthetase activity by post-translational lysine acetylation
Source: Front Mol Biosci. 2023 Mar 16;10:1117921. doi: 10.3389/fmolb.2023.1117921 (PMC10062202; doi:10.3389/fmolb.2023.1117921)

Supplemental Figure S3

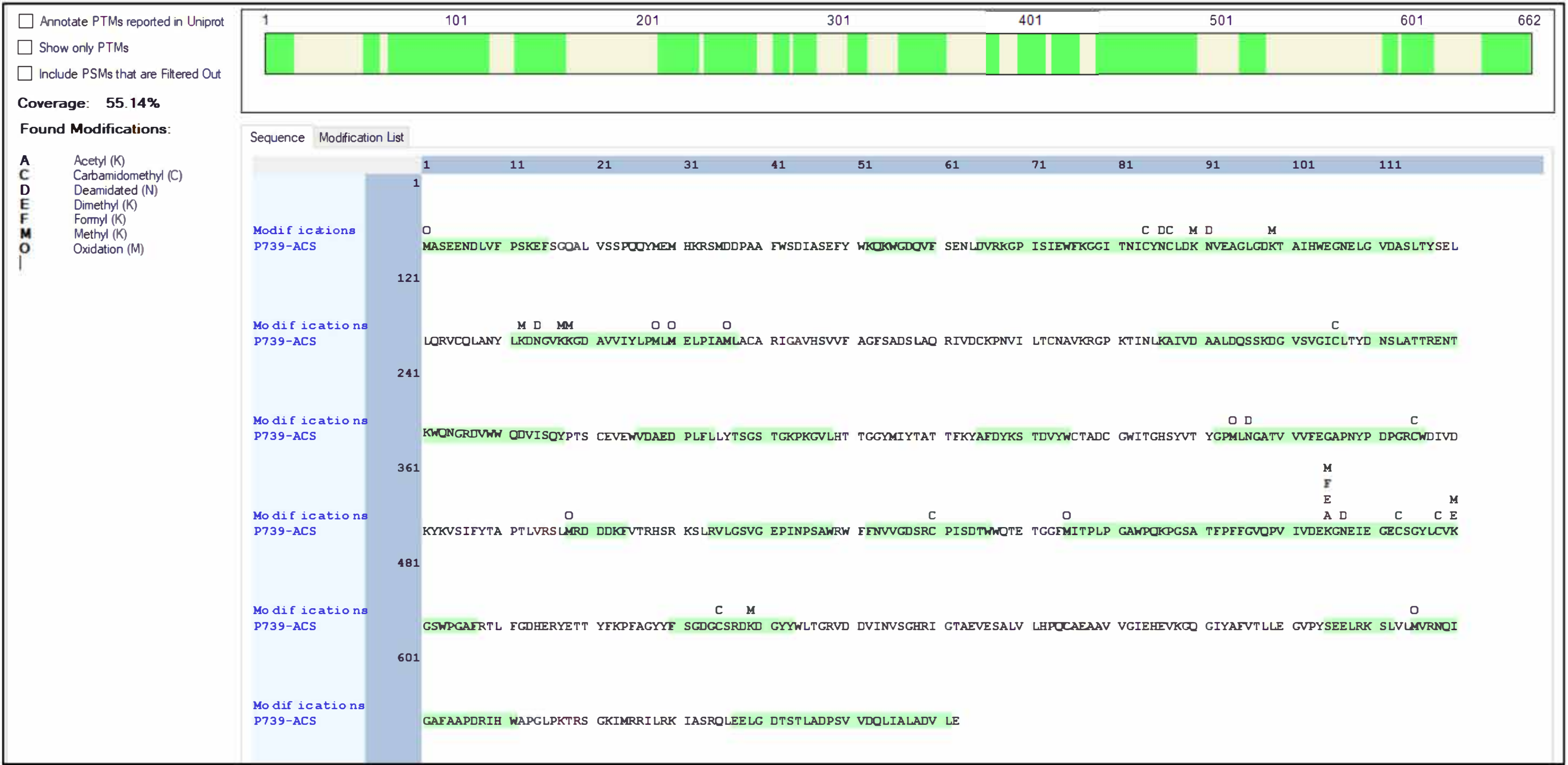

Supplement: Supplementary file 4 [file Image3.pdf]
